# Noninvasive monitoring of myocardial function after surgical and cytostatic therapy in a peritoneal metastasis rat model: assessment with tissue Doppler and non-Doppler 2D strain echocardiography

**DOI:** 10.1186/1476-7120-5-23

**Published:** 2007-07-12

**Authors:** Jens Hartmann, Fabian Knebel, Stephan Eddicks, Mark Beling, Andrea Grohmann, Alexander Panda, Christoph A Jacobi, Joachim M Müller, Klaus-Dieter Wernecke, Gert Baumann, Adrian C Borges

**Affiliations:** 1 Klinik für Allgemein-, Visceral-, Gefäß- und Thoraxchirurgie Charité Campus Mitte, Berlin, Germany; 2 Medizinische Klinik mit Schwerpunkt Kardiologie und Angiologie, Charité Campus Mitte, Berlin, Germany; 3 Yale University School of Medicine, Yale, USA; 4 Charité – Universitätsmedizin Berlin, Germany

## Abstract

**Objective:**

We sought to evaluate the impact of different antineoplastic treatment methods on systolic and diastolic myocardial function, and the feasibility estimation of regional deformation parameters with non-Doppler 2D echocardiography in rats.

**Background:**

The optimal method for quantitative assessment of global and regional ventricular function in rats and the impact of complex oncological multimodal therapy on left- and right-ventricular function in rats remains unclear.

**Methods:**

90 rats after subperitoneal implantation of syngenetic colonic carcinoma cells underwent different onclogical treatment methods and were diveded into one control group and five treatment groups (with 15 rats in each group): group 1 = control group (without operation and without medication), group 2 = operation group without additional therapy, group 3 = combination of operation and photodynamic therapy, group 4 = operation in combination with hyperthermic intraoperative peritoneal chemotherapy with mitomycine, and group 5 = operation in combination with hyperthermic intraoperative peritoneal chemotherapy with gemcitabine, group 6 = operation in combination with taurolidin i.p. instillation. Echocardiographic examination with estimation of wall thickness, diameters, left ventricular fractional shortening, ejection fraction, early and late diastolic transmitral and myocardial velocities, radial and circumferential strain were performed 3–4 days after therapy.

**Results:**

There was an increase of LVEDD and LVESD in all groups after the follow-up period (P = 0.0037). Other LV dimensions, FS and EF as well as diastolic mitral filling parameters measured by echocardiography were not significantly affected by the different treatments. Values for right ventricular dimensions and function remained unchanged, whereas circumferential 2D strain of the inferior wall was slightly, but significantly reduced under the treatment (-18.1 ± 2.5 before and -16.2 ± 2.9 % after treatment; P = 0.001) without differences between the single treatment groups.

**Conclusion:**

It is feasible to assess dimensions, global function, and regional contractility with echocardiography in rats under different oncological therapy. The deformation was decreased under overall treatment without influence by one specific therapy. Therefore, deformation assessment with non-Doppler 2D strain echocardiography is more sensitive than conventional echocardiography for assessing myocardial dysfunction in rats under oncological treatment.

## Background

Clinical echocardiography has been established as a safe, reproducible, and accurate assessment of cardiac anatomy, hemodynamics, and cardiac function. Commercially available ultrasound imaging systems are capable of resolution approaching that of magnetic resonance imaging as a result of recent computer and transducer technology. Understandably, there is increasing interest in using echocardiography as a basic research tool with standard laboratory animals. Knowledge of baseline normal values in commonly used rat species are limited [1,2]. Previous experimental studies could give some information about right and left ventricular contractility in sheep, pigs and dogs for with new technologies of 2D strain and tissue Doppler imaging [2]. Echocardiography has become widely used to evaluate cardiac function in animal models of cardiac diseases. Because of its non-invasive character, echocardiography allows serial in vivo evaluation of cardiac dimensions, ejection fraction, and diastolic parameters. New oncological complex therapeutic modalities have the potential to affect cardiovascular hemodynamics, preload, afterload, myocardial contractility, and may induce acute and chronic cardiac impairment. Therefore, these new therapy options have the potential to significantly alter echocardiographic parameters. There is limited knowledge about the impact of the combination of surgery, hyperthermia, and cytostatic therapy in sick rats. Knowledge of baseline echocardiographic (incl. tissue Doppler values and 2D echocardiography) normal values and changes under surgical and cytostatic therapy in an animal model with peritoneal metastasis would be worthwhile. This study was performed to assess the differential impact of different types and combinations of complex oncological therapy methods in rats with peritoneal metastasis.

## Methods

### Animal Preparation

All animal studies were performed in accordance with guidelines for the care and use of laboratory animals at our institutions. 90 male BDIX/HansHsd rats with a body weight between 227 and 312 g were obtained from a single breeding colony (Harlan Winkelmann, Borchen). Animals were individually housed and followed free access to standard laboratory food and water ad libitum and 12 hours of light per day. Maintance and care were carried out according to the guidelines of the local Animal Protection Commission. This protocol was approved by our local animal protection committee.

A new multimodal treatment system in tumor bearing rats was tested. For this purpose 2 × 10^5 ^syngenetic colonic carcinoma cells (DHD/K12/TRb) were implanted subperitoneal in right upper quadrant in male BDIX rats by a median mini-laparotomy under general i.p. anesthesia. After implantation rats were randomized into 6 groups (5 treatment groups, 1 control group). There were 15 rats in every group. 21 days after tumor implantation all animals of the treatment groups were operated by a standard median laparotomy under general i.p. anesthesia from 6 cm and tumor spread was analysed. After that, we created a surgical tumour debulking with the aim of complete tumour removal in all animals. 4 groups underwent an additional treatment like Hyperthermic Intraoperative Peritoneal Chemotherapy (HIPEC) with mitomycine (15 mg/m^2 ^body surface area) or with gemcitabine (24 mg/kg body weight) (group 1 and 2, respectively), Photodynamic Therapy (PDT) (group 3), taurolidine i.p. instillation (group 4). The rats of one group underwent only surgical debulking (group 5). 21 days after the surgical procedure all animals were sacrified in CO_2 _chamber and tumor weight, ascites volume and tumor spread, classified by a modified Peritoneal Cancer Index (PCI) were assessed by two independent observers.

The rats were anesthetized with xylazine (3.7 mg/kg i.p.) and ketamine (66.5 mg/kg i.p.), the anaesthesia was maintained throughout the echocardiographic examination, and electrocardiography was continuously monitored from limb leads. Animals were considered sufficiently anesthetized when they became totally unresponsive to a moderate pain stimulus while still normally breathing spontaneously (absence of respiratory depression, animals were not intubated). The anterior chest was shaved and the rats were postioned in left lateral decubitus position during image acquisition.

### Echocardiographic examination

Echocardiographic studies were performed (VIVID 7 dimension system; General Electric-Vingmed Ultrasound, Horton Norway). Images were obtained using a 10S transducer (5.5–12 MHz) with high temporal and spatial resolution. The transducer was placed directly on the chest wall. A complete 2-dimensional, M-mode (according to standards of American Society of Echocardiography), and color and tissue Doppler echocardiogram was performed under anesthesia.

Using a zoomed image window, color Doppler myocardial velocity data were acquired at a frame rate of 205–230 frames/s, a sector angle of 30 degrees, and a image depth of 15 mm. Beam focus was set at 10 mm. Digital data of 5 consecutive heart cycles were recorded and transferred to a personal computer workstation for offline analysis.

Circumferential 2D strain was calculated from the parasternal short axis view (anterior septal and inferior mid wall). For radial contractility measurements, strain profiles were analyzed on the inferior and anterior myocardium. Peak systolic strain values were measured in each of 5 heart cycles. The maximal and minimal values were discarded, and the remaining 3 values were averaged. We did not measure strain rate with the speckle tracking method because of the too low temporal resolution and the smoothing of the curves of this method.

Systolic 2-dimensional strain (reflects relative longitudinal shortening = negative values, and longitudinal elongation or stretching = positive values, compared with end diastole in %), and strain rate (reflects the velocity of strain change) were calculated with the new software for echocardiographic quantification as previously described, based on real-time tracking of natural acoustic marker during two consecutive frames by 2-dimensional strain software. In addition, myocardial velocities with estimation of longitudinal displacement (systolic time velocity integral), strain, strain rate, and acceleration during isovolumic contraction were measured in the same segments with conventional TDE [3].

Images and offline reconstructed 2D strain images were compared for each individual rat and interpreted by two investigators, blinded to experimental information and timing of the studies. 2D strain and strain rate were calculated as previously described [4].

### Data analysis

The RV morphology was assessed as RV end-diastolic diameter (RVEDD). To assess RV function, the base-to-apex shortening during systole, measured as the tricuspid annular plane systolic excursion (TAPSE) of the lateral portion of the tricuspid annular plane, was recorded in the M-mode format under 2D echocardiographic guidance from the apical 4-chamber view.

Radial and longitudinal myocardial strain rate and strain were calculated from color Doppler velocity data using special software (Echo Pac, GE Medical Systems) as previously described [5].

For analysis, rats divided into two groups: group A with potential cardiotoxic therapy (group 1 and 2) and group B with non-cardiotoxic therapy (group 3–5).

### Statistical analysis

Values are expressed as mean ± standard deviation (SD) unless indicated otherwise. Groups were compared by parametric or non-parametric tests (t-tests and Wilcoxon-Mann-Whitney tests, resp.). More than 2 groups were analysed using ANOVA (symmetrically distributed observations) or Kruskal-Wallis test (otherwise). Post-hoc tests were performed (if significant differences were proved globally) with the help of multiple tests or pair-wise comparisons (with the same error of the 1^st ^kind in 3 groups – closed test procedure). Categorical data were tested by means of Fisher's exact test, in particular differences in sensitivity, specificity and accuracy. Proceeding on the assumption that SR measurements are independent from tethering effects of adjunctive segments we choose the segment as statistical unity in the corresponding tests. Simple linear regression, intra-class correlation and Kappa were used for measurement comparisons.

Because of the fact that the calculated p-values are to be understood as explorative ones, no adjustments for multiple testing were made. Additionally, no generalisation of the results can be undertaken, particularly not in the sense of superiority.

## Results

### Feasibility

Echocardiographic measurements were possible in all rats before and after treatment. All animals survived and we did not encounter any hemodynamic or respiratory instability or arrhythmias during sedation, examination, oncological treatment or follow-up.

### Effects of oncological treatment

The heart rate after follow-up was higher in the control group (235 ± 34 vs 274 ± 47 bpm; P = 0.006), whereas it remained unchanged in rats of the treatment group (groups 2–6) 231 ± 17 vs 185 ± 27 bpm; P = 0.62).

**Figure 1:**
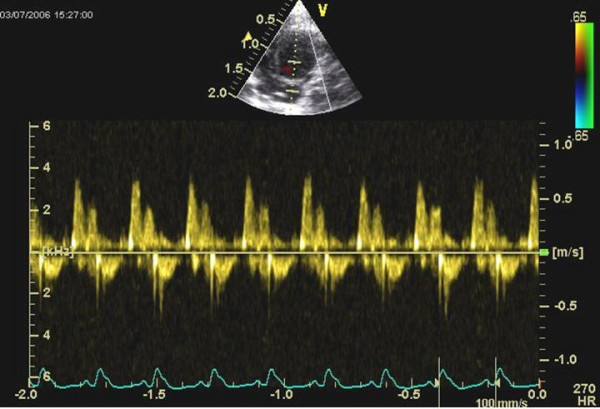
Pw-Doppler measurement of the transmitral flow velocity obtained from the apical view.

**Figure 2:**
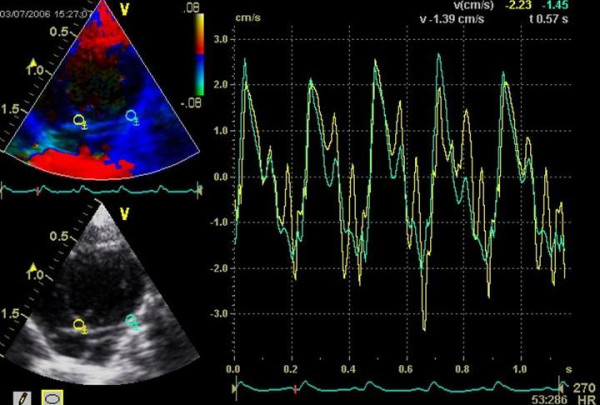
Tissue Doppler measurement from the apical view with calculation of systolic and diastolic velocities of the basal segments (septum and lateral wall).

**Figure 3:**
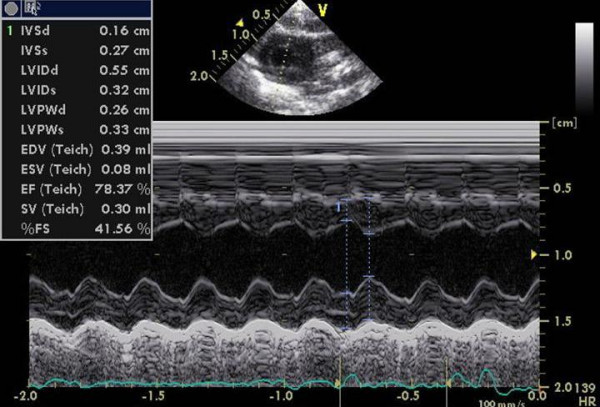
M-Mode from the short axis view with example of calculation of the LV dimensions at baseline.

**Figure 4:**
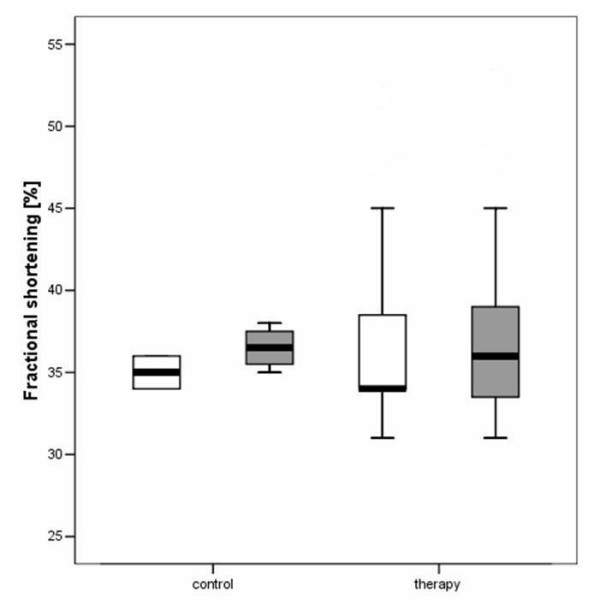
Boxplot analysis of fractional shortening (FS) in the control and therapy group. The white boxes: before; grey boxes: after therapy.

**Figure 5:**
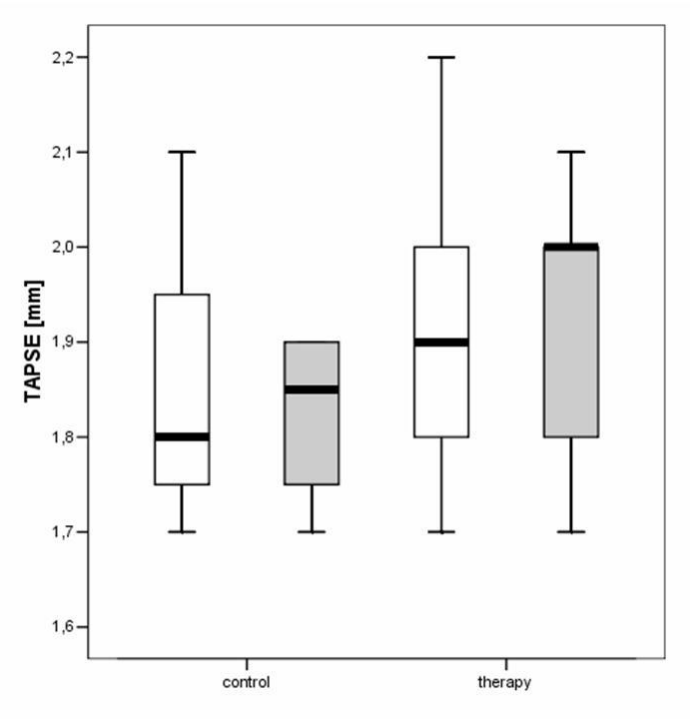
Boxplot analysis of tricuspid annular plane systolic excursion (TAPSE) in the control and therapy group. The white boxes: before; grey boxes: after therapy.

**Figure 6:**
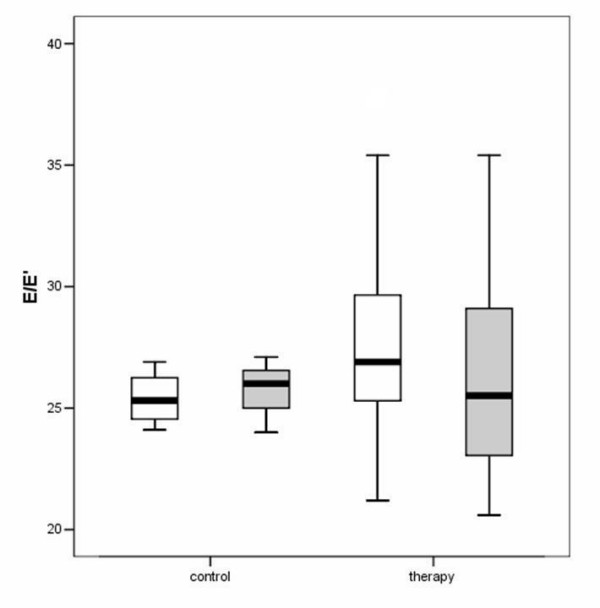
Boxplot analysis E/E' in the control and therapy group. The white boxes: before; grey boxes: after therapy.

**Figure 7:**
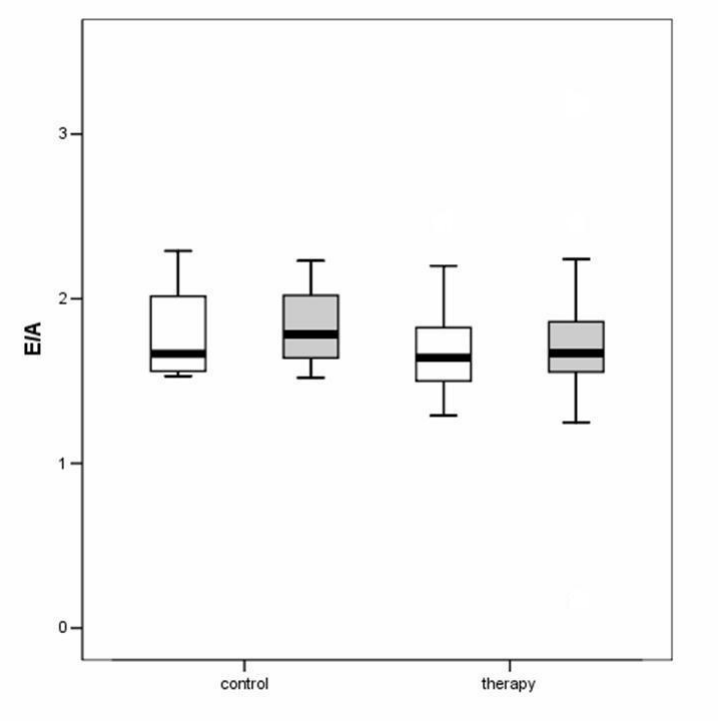
Boxplot analysis of E/A in the control and therapy group. The white boxes: before; grey boxes: after therapy.

The hemodynamic effects in normal animals as well as in the different treatment groups are summarized in Table 1, 2, 3. There was an increase of LVEDD and LVESD in all groups after the follow-up period (P = 0.0037). Other LV dimensions, FS and EF measured by echocardiography were not significantly affected by the different treatments. Diastolic mitral filling parameters and myocardial velocities were not significantly affected by the type of treatment (figures 1+2). Values for right ventricular dimensions and function (TAPSE) remained unchanged under treatment and there were no differences between the groups (see figures 1, 2, 3, 4, 5, 6, 7)

**Table 1:** Characteristics and echocardiographic data of the study animals

	Group 2–6 *before/after therapy*	Group 1	Group 2	Group 3	Group 4	Group 5	Group 6
Body mass – g							
Mean	275	282	267	288	269	275	274
SD	23	26	24	25	24	20	23
Range	227–312	258–318	227–287	247–312	235–306	237–291	235–312
Heart rate – bpm							
Mean	263/261	265/274*	260/253	277/260	260/253	260/265	259/268
SD	17/27	11/11	21/29	12/22	20/38	18/23	14/27
Range	231–289/185–311	223–249/215–287	244–288/221–296	256–288/234–298	235–289/185 – 289	245–289/234 – 300	231–278/221–311
Enddiastolic diameter-mm							
Mean	7/7.2*	6.9/7.2*	6.7/7.1*	7.6/7.8	6.6/6.8	7.1/7.3	6.8/7.1
SD	0.7/0.6	0.64/0.62	0.67/0.43	0.29/0.53	0.17/0.84	0.25/0.30	0.7/0.4
Range	5.0–8.1/5.5–8.7	6.3–7.8/6.5–7.9	6.0–7.8/6.4 – 7.6	7.3–8.1/7.1 – 8.7	5.0–7.4/5.5 – 7.3	6.8–7.6/6.9–7.8	5.4–7.6/6.5–7.9
Endsystolic diameter- mm							
Mean	2.8/3.1*	2.7/3.0*	2.6/3.0*	2.7/3.3	2.5/2.7	2.6/2.8	2.5/2.7
SD	0.6/0.7	0.6/0.67	0.56/0.65	0.54/0.62	0.4/0.45	0.37/0.34	0.34/0.27
Range	2.1–4.1/2.1–4.0	2.2 – 4.0/2.3–4.1	2.3 – 4.1/2.4 – 4.2	2.5–4.1/2.6–4.0	2.2–4.0/2.3–4.1	2.3 – 4.1/2.2–4.1	2.3–4.0/2.3–4.1
Enddiastolic septal wall-mm							
Mean	1.3/1.3	1.3/1.3	1.3/1.2	1.2/1.2	1.6/1.5	1.2/1,2	1.4/1.4
SD	0.20/0.22	0.14/0.09	0.3/0.11	0.08/0.10	0.23/0.17	0.08/0.05	0.33/0.33
Range	1–2.0/1.1–2.0	1.2–1.5/1.2–1.4	1.1–1.8/1.1–1.4	1.1–1.3/1.1–1.4	1.3 – 2.0/1.2 – 1.7	1.1–1.3/1.1 – 1.2	1.0 – 1.9/1.1–1.4
Enddiastolic inferior wall-mm							
Mean	1.4/1.4	1.22/1.3	1.48/1.28	1.25/1.30	1.6/1.6	1.2/1.2	1.4/1.5
SD	0.3/0.3	0.09/0.14	0.2/0.1	0.10/0.12	0.41/0.51	0.13/0.082	0.27/0.38
Range	1.1–2.3/1.1–2.6	1.1–1.3/1.1–1.4	1.2 – 2.2/1.1.-1.4	1.1–1.4/1.1–1.4	1.2 – 2.3/1.2 – 2.6	1.1–1.4/1.1 – 1.3	1.1–1.9/1.2–2.3
Fractional shortening-%							
Mean	36.6/37.1	35.0/36.5	36.2/35.0	33.0/33.8	40. 0/43.2	34/35	39/38
SD	4.9/5.2	1.5/1.5	3.5/2.7	1.8/2.2	5.2/6.1	2.5/2.3	6.3/5.5
Range	31–52/31–53	34–36/35–38	34–42/32–39	32–34/31–37	34–47/37–53	31–37/32 – 38	32–52/31–45
Left ventricular ejection fraction-%							
Mean	73/74	71/72	71/70	74/74	75/78	71/72	74/74
SD	5/4	2/4	5/3.5	2.5/2.1	6.7 – 6.9	1.7/2.5	7.3/4.9
Range	62–87/66–88	68–73/66 – 75	67 – 79/66–75	71–78/71–76	67 – 83/70 – 88	68 – 73/69–74	62–87/67–81
Right ventricular enddiastolic diameter-mm							
Mean	3.4/3.5	3.5/3.6	3.4/3.6	3.4/3.7	3.34/3.45	3.3/3.5	3.2/3.4
SD	0.16/0.14	0.14/0.13	0.22/0.21	0.21/0.23	0.16/0.21	0.21/0.22	0.19/0.22
Range	3.3 – 4.0/3.5 – 4.1	3.4–4.1/3.4 – 4.3	3.3–4.0/3.2 – 4.3	3.4–4.1/3.3 – 4.3	3.4–4.1/3.2–4.3	3.2–3.9/3.4 – 4.1	3.4 – 3.9/3.4 – 4.1
Tricuspid annular plane Systolic excursion (TAPSE)-mm							
Mean	1.9/1.9	1.85/1.82	1.94/1.86	1.88/1.95	1.88/1.95	1.9/1.9	1.91/1.92
SD	0.12/0.11	0.17/0.10	0.16/0.11	0.13/0.08	0.13/0.09	0.11/0.09	0.09/0.14
Range	1.7 – 2.2/1.7–2.1	1.7 – 2.1/1.7 – 1.9	1.8–2.2/1.7–2.0	1.7–2.1/1.8–2.0	1.7–2.1/1.8–2.0	1.7–2.0/1.8–2.0	1.8–2.0/1.7–2.1

Group 1 = control group (without operation and without medication)

Group 2 = operation group without additional therapy

Group 3 = combination of operation and photodynamic therapy

Group 4 = operation in combination with hyperthermic intraoperative peritoneal chemotherapy with mitomycine

Group 5 = operation in combination with hyperthermic intraoperative peritoneal chemotherapy with gemcitabine

Group 6 = operation in combination with taurolidin i.p. instillation;

* P < 0.05; ** P < 0.001

**Table 2:** Diastolic function parameters

	Group 2–6 *Before/after therapy*	Group 1	Group 2	Group 3	Group 4	Group 5	Group 6
E-wave – cm/s							
Mean	76/75	73/76	71.8/71.6	78/78.7	78/70.3	75.8/72.3	75.6/79.0
SD	7.6/10.2	4.2/2.8	4.0/4.0	8.3/8.2	10.5/10.1	7.6/8.6	7.1/14.1
Range	66 – 98/57–101	69 – 78/72–78	66–76/68–78	68–88/69–88	69–98/57–87	69–88/66–89	68–87/67–101
A-wave- cm/s							
Mean	45/52	42/42	43.6/44.4	47.3/45.7	45/44	46/45	45.2/47.3
SD	5.0/6.4	5.1/6.1	5.9/6.2	6.3/8.3	4/3.7	4.8/2.7	5.1/5.4
Range	35 – 57/31 – 64	34–45/35 – 50	35–50/35–51	40–56/34–56	40–50/43–45	41–51/41–49	41–57/31–48
E/A ratio							
Mean	1.69/1.69	1.78/1.83	1.67/1.63	1.68/1.76	1.77/1.59	1.66/1.60	1.68/1.81
SD	0.27/0.47	0.34/0.29	0.23/0.21	0.33/0.33	0.36/0.23	0.25/0.22	0.21/0.18
Range	1.29 – 2.45/1.23 – 2.3	1.53–2.29/1.52–2.23	1.38–2.0/1.38–1.94	1.29–2.20/1.25–2.24	1.4–2.45/1.27–1.98	1.45–2.15/1.35–1.98	1.44–2.0/1.34–1.98
Mitral inflow deceleration time – msec							
Mean	48.6/48.2	50.2/50.0	47.0/48.2	46.8/46.3	47.7/47.6	50.8/49.3	49.8/49.3
SD	3.4/3.2	4.7/4.5	2.5/4.9	3.1/2.58	2.6/2.1	3.7/2.5	3.5/3.1
Range	43–56/43–55	46 – 57/45–56	44–50/43–55	43–52/44–50	43–50/45–50	45–55/47–53	46–56/45–54
Tissue Doppler E'-cm/s							
Mean	2.8/2.9	2.87/2.95	2.74/2.80	2.65/3.02	2.8/2.8	2.78/2.75	2.75/2.91
SD	0.28/0.27	0.12/0.10	0.22/0.38	0.41/0.18	0.23/0.36	0.22/0.25	0.28/0.17
Range	2.2 – 3.4/2.3 – 3.4	2.7–3.9/2.3 – 3.4	2.4–3.0/2.4 – 3.4	2.2–3.4/2.8 – 3.3	2.6–3.1/2.3–3.4	2.4–3.0/2.4–3.0	2.32–3.1/2.7–3.2
Tissue Doppler A'-cm/s							
Mean	2.1/2.1	2.1/1.73	2.12/2.22	2.3/2.3	2.16/1.88	2.23/2.23	1.89/1.96
SD	0.3/0.31	0.31/0.26	0.34/0.31	0.08/0.19	0.41/0.34	0.12/0.16	0.29/0.37
Range	1.49 – 2.90/1.44–2.6	1.7–2.4/1.5 – 2.0	1.6–2.5/1.8 – 2.6	2.2–2.4/2.0–2.5	1.6–2.9/1.5–2.3	2.1–2.4/2.0–2.4	1.49–2.4/1.44–2.4
E to E' ratio							
Mean	27.8/26.2	25.4/25.8	26.3/25.9	30.1/26.2	27.7/25.0	27.3/26.5	27.6/27.1
SD	3.7/3.8	1.17/1.29	2.25/3.99	6.18/3.33	2.2/3.3	3.0/4.5	3.5/4.7
Range	21.2 – 37.7/20.6–35.4	24.1–26.9/24.0–27.1	24.4–30.0/20.6–30.4	21.2–37.7/20.9 – 29.3	26.3–31.8/21.1–30.4	23 – 30.8/22.3–34.2	23.4–34.2/22.9–35.4

Group 1–6 (see Table 1)

**Table 3:** Radial and circumferential 2D strain

	Group 2–6 *Before/after therapy*	Group 1	Group 2	Group 3	Group 4	Group 5	Group 6
*Radial *Strain							
Anterior Strain-%							
Mean	21.6/22.4	24.2/22.7	20.6/21.4	22.6/21.6	22.1/22.8	21.8/22.1	21.0/23.5
SD	4.0/4.0	2.9/1.7	2.4/1.8	3.0/3.6	8.5/6.2	1.5/1.3	1.9/5.2
Range	12.6–38.0/18.0–36.0	21–28/21–25	18–24/19–24	19–27/18–28	12.6–38.0/18–35	19–23/20–24	18–24/20–36
Inferior Strain – %							
Mean	20.0/19.4	21.2/18.5	20.4/19.8	20.5/18.8	21.6/22.5	19.8/18.5	18.4/18.1
SD	4.3/4.1	2.2/2.6	2.3/2.9	4.5/1.3	6.7/7.5	4.1/2.1	3.5/3.2
Range	11–35/11–37	18–23/16–22	17–22/15–22	17–29/17–21	17–35/16–37	12–23/16–22	11–24/11–22
*Circumferential *strain							
Anterior Strain-%							
Mean	-22.1/-22.6	-22.0/-22.0	-22.0/-21.6	-22.0/-21.8	-22.3/23.0	-21.5/23.8	-22.4/-22.8
SD	2.5/2.5	3.6/1.6	2.3/3.4	0.89/2.7	3.1/2.9	2.8/1.9	3.2/2.0
Range	17–26.6/18–27	19–26/20–24	18–24/18–26	21–23/18–25	18–25/19–27	19–25/21–26	17–26.6/19–25
Inferior Strain-%							
Mean	-18.1/-16.2*	-18.0/-17.2	-17.8/-16.2	-16.8/16.3	-17.6/-16.8	-18.3/-16.3	-19.6/-15.7
SD	2.5/2.9	2.2/3.3	1.6/3.6	0.76/1.2	2.1/2.8	1.7/1.5	3.8/4.5
Range	14–25/9–22	16–21/14–21	16–19/12–22	16–18/15–18	15–21/13–21	16–21/14–18	14–25/9–22

Group 1–6 (see Table 1)

### 2D strain echocardiography measurements

The changes of values for radial and circumferential contraction were summarized in Table 3. There are heterogeneous values of circumferential strain: circumferential strain baseline values of the anterior wall were higher compared with values of the inferior wall in all animals: 22.0 ± 2.2 vs 17.5 ± 3.1%; P = 0.001).

**Figure 8:**
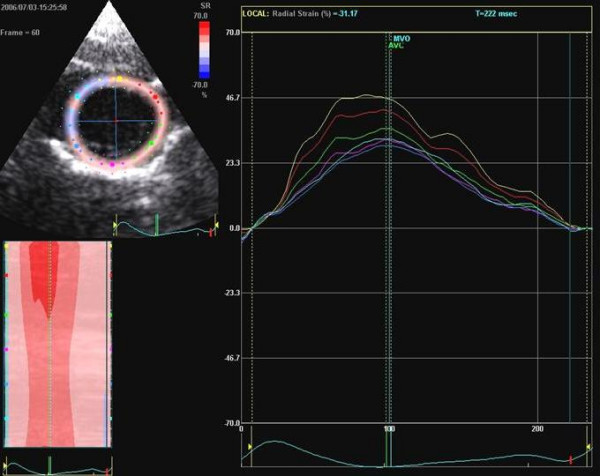
Non-Doppler 2D strain measurements of the radial deformation of the anterior and inferior wall at baseline.

In all rats after treatment, the strain profiles demonstrated a postsystolic thickening pattern with circumferential strain in the inferior wall segment, but not in the control group (figures 8+9).

**Figure 9:**
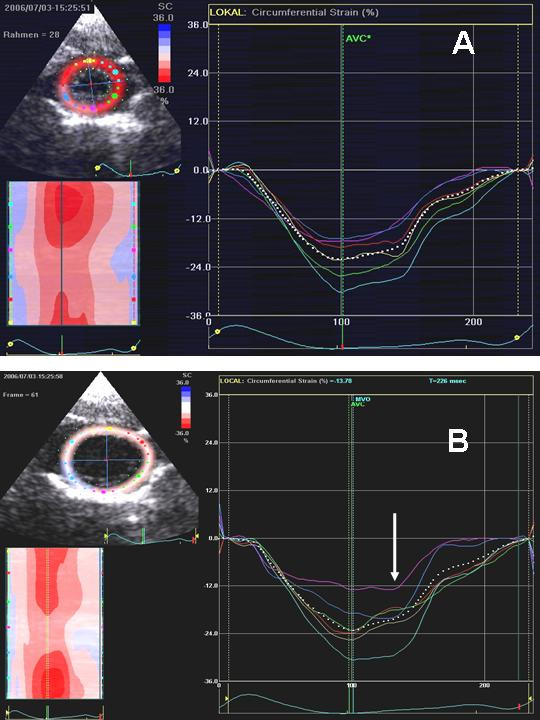
**A and B **Non-Doppler 2D strain measurements of the circumferential deformation of the anterior and inferior wall at baseline (A) and after treatment with reduction of the maximal strain und postsystolic shortening (arrow) in the inferior wall.

**Figure 10:**
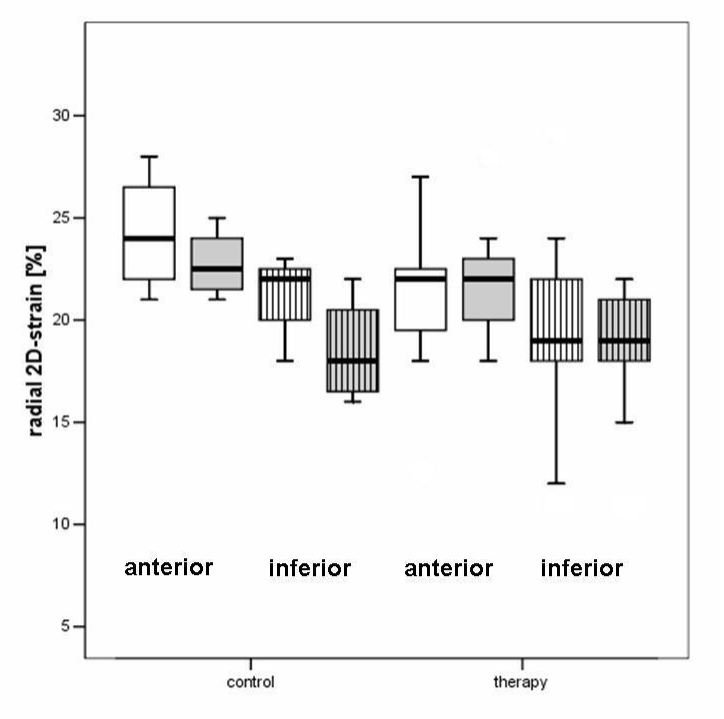
Boxplot analysis of non-Doppler radial 2-D strain in the control and therapy group. The white boxes: before; grey boxes: after therapy. The plain boxes represent the anterior, the striped boxes the inferior 2-D strain

**Figure 11:**
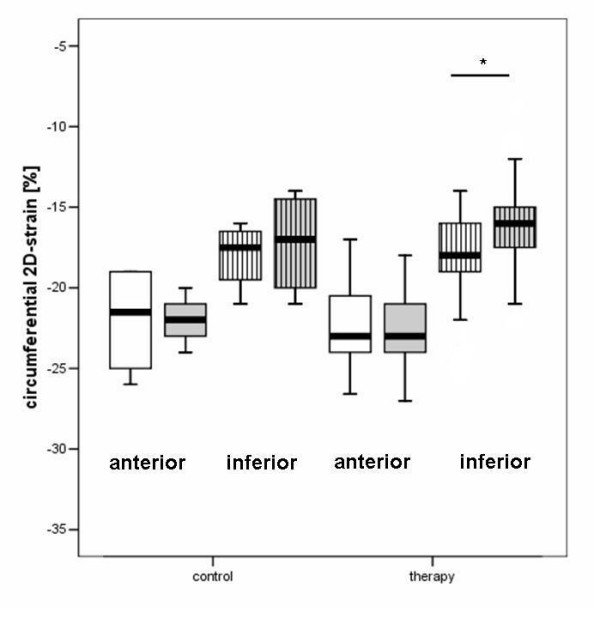
Boxplot analysis of non-Doppler circumferential 2-D strain in the control and therapy group. The white boxes: before; grey boxes: after therapy. The plain boxes represent the anterior, the striped boxes the inferior 2-D strain.

Radial strain parameters were not significantly affected by the type of treatment (Table 3). Circumferential strain of the inferior wall was reduced after treatment in group 6 (P = 0.02); and compared with the group 1 (controls) the circumferential strain values were reduced after follow-up in the overall treatment group (group 1: -18.0 ± 2.1 before and -17.25 ± 3.3% after vs group 2–6: -18.1 ± 2.5 before and -16.2 ± 2.9% after; P = 0.001). Circumferential strain between the single treatment groups were not significant different (table 3, figures 10, 11).

## Discussion

This is the first study demonstrating the feasibility of 2D strain echocardiography in rats for noninvasive quantification of regional ventricular function before and after oncological treatment. We could show that conventional systolic and diastolic parameters remained unaffected by the treatment, whereas circumferential 2D strain of the inferior wall was slightly, but significantly reduced after the treatment without differences between the single treatment groups.

Previous studies with animal models have examined a wide variety of cardiac diseases and therapies with a trend from invasive to non-invasive hemodynamic assessment in recent years.

M-mode and 2D echocardiography are adequate in the absence of regional wall-motion abnormalities. However, the small size of the rat heart and the relatively fast heart rate preclude accurate measurements in case of regional wall-motion abnormalities Previous studies demonstrated that the myocardial velocity gradient derived from Doppler tissue imaging was impaired in rats with pressure overload-induced left ventricular hypertrophy [6]. Strain and strain rate (SR) are useful for quantification of changes and spatial distribution of regional contractile function in rats [7].

Cytostatic agents, surgical procedures, and anesthetic agents are known to have effects on myocardial function. Therefore, the choice of anesthetic agent and the potentially cardiotoxic therapy have the potential to affect echocardiographic data directly [8]. Mitomycine and gemcitabine have the potential for a decrease of contractility and direct cardiotoxic effects. Xylazine, however, is considered an clonidine analog, which can result in hypotention and bradycardia, i.e. counteracting the global cardiovascular effects of ketamine. Ketamine has been previously shown in animal models to negatively affect diastolic function [9]. However, these studies were conducted in the presence of pharmacologic blockade of the autonomic nervous system, which was not the case on our rats. In our study, LV dimensions, EF, and diastolic function were not significantly affected by the sedation or surgical plus oncological therapy.

It was assumed that left-ventricular long-axis function may be more sensitive to ischemia than short-axis function, others could demonstrate that circumferential strain reduction and disorders in twisting are very early signs of myocardial damage in some entities [10,11]. Serri et al. demonstrated that 2D strain echocardiography identified early, subclinical global systolic dysfunction in patients with hypertrophic cardiomyopathy [11]. Thus, TDI and 2D strain echocardiographic data are complementary to short axis scans (indicating wall thickening) and to non-contrast harmonic imaging echocardiography (endocardial border detection).

In both *in vitro *and *in vivo *models, non-Doppler 2D strain echocardiography demonstrated a good correlation and agreement with sonomicrometry values under different contractile conditions [12,13]. Strain and SR measurements that are obtained by the non-Doppler 2D strain echocardiograpahy correlate well with tissue Doppler-derived measurements [5]. Compared to TDI based echocardiography, non TDI-2D-strain seems to have a particularly low inter- and intraobserver variability [12,14]

TDI- and non Doppler-2D strain echocardiography permit quantitative assessment of global and regional ventricular function [12] and could therefore improve the diagnostic accuracy especially for the inexperienced observer to detect regional wall motion abnormalities. Hirano et al. [7] found that absolute peak systolic strain values were consistently 20% lower than percent wall thickening. This difference could result from the calculation distance of SR used in their study, which might not have been adapted to the rat heart size. The assessment of percent change in SR and strain remains useful.

In contrast to other studies, we could not see postsystolic thickening or systolic wall thinning, under ischemic conditions [7].

Our findings regarding a possible temporary disturbance of the LV torsion (decreased circumferential deformation) due to possible afterload mismatch is in accordance to first human findings indicating reduced and delayed diastolic untwisting with aging [10], and uniformly decreased segmental LV torsion in patients with amyloid-related cardiomyopathy measured with 2D strain echocardiography [11].

## Conclusion

In conclusion, our results demonstrate a high technical reproducibility and diagnostic accuracy with both good spatial and temporal resolution of conventional 2D-, TDI- and Non-Doppler 2D strain echocardiography.

We conclude that the demonstrated negative effect on circumferential strain of the inferior wall was a general effect of the sedation plus oncological therapy and not induced by one special modality of therapy. Deformation assessment with non-Doppler 2D strain echocardiography is more sensitive than conventional echocardiography for assessing myocardial dysfunction in rats under oncological treatment.

Non-Doppler 2D strain echocardiography represents a new, powerful method for the evaluation and quantification of global and regional myocardial function for experimental *in vivo *protocols in small animals.

## Limitations

The methods used in this study were not compared to a gold standard technique (e.g. MRI, sonomicrometry) as these methods were not part of the protocol.

The heart rate in the animals was greater than 300/min, even under anesthetic conditions. The frame rate had to be set between 180 and 210 for tissue Doppler measurements and between 60 and 80 for non-Doppler 2D strain calculations, in order to balance the requirements for temporal and spatial resolution.

Deformation measurement is limited in the short axis views due to twisting and swinging in the azimuthal plane of the heart and the movement by respiration. We preferred these views for deformation calculation because of the difficulty in acquiring good apical images as previously described [7].

Histological examinations and follow-up examinations were not performed. We can only speculate about the temporary character of the changes of circumferential strain values in the treatment group.

## Abbreviations

LV = left ventricular

ECG = electrocardiogram

pw- Doppler = pulsed wave Doppler

LVEF = left ventricular ejection fraction

2D = two-dimensional

TDI = tissue Doppler imaging

SR = strain rate

ROI = region of interest

## Competing interests

The author(s) declare that they have no competing interests.

## Authors' contributions

JH, FK and ACB have designed and performed the study and have written the manuscript. JH and FK have equally contributed to this study. JH, FK, ACB SE, MB, AG, AP, GB have performed echocardiographic measurements and participated in the study design and coordination. JH, CJ and JM have performed the oncological and surgical therapy. KW has supported the statistical analysis of the study. All authors have read and approved the final manuscript.
